# Health Problems and Associated Factors Among Grain Handlers in Bangladesh: A Cross-Sectional Study

**DOI:** 10.7759/cureus.110849

**Published:** 2026-06-14

**Authors:** Naima Ferdous, Mohammad Ataour Rahman, Aparna Mandal Anee, Nishat Jahan Nishi, Md Faizul Ahasan, Ahad Mahmud Khan, Shahria Sattar

**Affiliations:** 1 Department of Occupational and Environmental Health, National Institute of Preventive and Social Medicine, Dhaka, BGD; 2 Research and Development Unit, Dhaka Bank Public Limited Company, Dhaka, BGD; 3 Department of Pharmacology and Therapeutics, Ibrahim Medical College, Bangladesh Institute of Research and Rehabilitation in Diabetes, Endocrine and Metabolic Disorders General Hospital, Dhaka, BGD; 4 Department of Public Health, Projahnmo Research Foundation, Dhaka, BGD

**Keywords:** bangladesh, grain handlers, musculoskeletal problems, occupational health, respiratory problems, rice mill workers

## Abstract

Background

Grain handlers are exposed to several occupational hazards, including grain dust, heat, smoke, heavy physical workload, prolonged working hours, and repetitive movements. These exposures may increase the risk of respiratory, musculoskeletal, eye, skin, and gastrointestinal problems. This study aimed to assess health problems and associated factors among grain handlers in Bangladesh.

Methods

A cross-sectional study was conducted among 292 grain handlers working in rice mills in Munshiganj Sadar Upazila, Dhaka Division, Bangladesh, in 2021. Data were collected through face-to-face interviews using a semi-structured questionnaire. Socio-demographic characteristics, work-related factors, and self-reported health problems were assessed. Descriptive statistics were used to summarize the findings. Bivariable and multivariable logistic regression analyses were performed to identify factors associated with health problems.

Results

The mean age of the respondents was 38.04±11.01 years, with a male-to-female ratio of 5:1. Most workers worked >48 hours per week (86.3%) and reported overtime work (86.3%). Respiratory problems were the most common health problem, reported by 227 workers (77.7%), followed by musculoskeletal problems in 189 (64.7%), eye problems in 73 (25.0%), gastrointestinal problems in 49 (16.8%), and skin problems in 48 (16.4%). The most common symptoms were cough in 203 (69.5%), lower back pain in 107 (36.6%), red eye in 65 (22.3%), itching in 48 (16.4%), and abdominal pain in 29 (9.9%). In the adjusted analysis, no factor was significantly associated with respiratory problems. Eye problems were less likely among workers in the boiling section than among those in the drying section (AOR=0.40, 95% CI: 0.17-0.96; p=0.039). Primary education was associated with lower odds of skin problems compared with no formal education (AOR=0.39, 95% CI: 0.17-0.88; p=0.024). Musculoskeletal problems were significantly more common among workers aged 39-58 years than among those aged 19-38 years (AOR=2.71, 95% CI: 1.35-5.44; p=0.005) and among loading/unloading workers compared with drying workers (AOR=2.23, 95% CI: 1.10-4.53; p=0.026). Gastrointestinal problems were more likely among workers working >48 hours per week than among those working ≤48 hours per week (AOR=4.05, 95% CI: 1.16-14.15; p=0.029).

Conclusion

Grain handlers in Bangladesh experience a high burden of occupational health problems, particularly respiratory and musculoskeletal problems. Age, education, section of job, and weekly working hours were associated with selected health problems. Because of the cross-sectional design and reliance on self-reported outcomes, these findings should be interpreted as associations rather than causal effects. Improved occupational safety measures, including dust control, ventilation, ergonomic interventions, scheduled rest periods, health education, personal protective equipment, and regular health screening, are needed to protect this vulnerable workforce.

## Introduction

Grain handling is an important occupational activity in Bangladesh, where rice is the principal staple food and rice processing provides employment for a large number of unskilled and semi-skilled workers [1]. In rice mills, grain handlers are involved in different stages of rice processing, including receiving, carrying, drying, boiling, husking, milling, sorting, packaging, loading, and unloading grains. These activities are often performed in small- or medium-scale mills where occupational safety practices may be limited [2]. Consequently, workers may be exposed to grain dust, heat, smoke, noise, heavy physical workloads, awkward postures, and repetitive movements [3].

Grain dust is a complex mixture of organic and inorganic materials, including particles from grain, husk, bran, silica, fungi, bacteria, endotoxins, insects, mites, animal waste, and chemical residues. Workers exposed to grain dust may develop respiratory symptoms such as cough, shortness of breath, chest tightness, runny nose, wheezing, and other chronic respiratory complaints. Dust exposure may also cause eye irritation, redness, watering, and a burning sensation. In addition, contact with grain dust and related materials can lead to skin problems such as itching and rashes [4-7].

Besides dust-related problems, grain handlers are also exposed to substantial ergonomic hazards. Carrying heavy sacks, lifting loads, prolonged standing, bending, repetitive hand movements, and working long hours may increase the risk of musculoskeletal problems, including neck pain, shoulder pain, back pain, knee pain, and ankle or foot discomfort. Gastrointestinal symptoms and accidental injuries may also occur due to poor working conditions, inadequate rest, lack of workplace hygiene, and limited access to occupational health services [8,9].

In Bangladesh, occupational health and safety among informal and semi-formal workers remains a neglected area. Grain handlers often belong to low socio-economic groups, have limited formal education, and may have poor awareness of occupational health risks. Inadequate or inconsistent use of personal protective equipment, long working hours, overtime work, smoking, poor ventilation, and lack of routine health screening may further increase their vulnerability [10]. Although rice milling and grain handling are common occupations in Bangladesh, evidence on the burden of health problems and associated factors among grain handlers remains limited.

Understanding the pattern of health problems among grain handlers is important for planning workplace interventions, improving occupational safety practices, and protecting the health of this vulnerable workforce. Therefore, this study aimed to assess health problems among grain handlers in Bangladesh and identify socio-demographic and work-related factors associated with these health problems.

## Materials and methods

Study design and setting

This cross-sectional study was conducted among grain handlers working in rice mills in Munshiganj Sadar Upazila, Bangladesh, in 2021. The study included grain handlers from different sections of the rice mills, including drying, boiling, husking, and loading/unloading.

Study population

The study population included grain handlers aged 19 years and above who had been working in rice mills for at least six months. Workers involved in receiving, handling, storing, boiling, drying, husking, loading, unloading, or processing grains were considered eligible. Workers who were unwilling to participate or unable to provide informed consent were excluded.

Sample size and sampling

The sample size was calculated using the single population proportion formula, considering a 95% confidence level, an assumed 50% prevalence of health problems due to limited prior evidence, and a 6% margin of error. The calculated sample size was 267. After adding approximately 10% for non-response or incomplete data, the required sample size was 294. Finally, 292 grain handlers were included in the study.

Data collection procedure

Data were collected through face-to-face interviews using a semi-structured questionnaire. The questionnaire was developed after reviewing relevant occupational health literature and included information on socio-demographic characteristics, work-related conditions, and self-reported health problems. Socio-demographic variables included age, gender, religion, educational qualification, marital status, number of family members, type of house, monthly income, and smoking status. Work-related variables included duration of work experience, section of job, weekly working hours, and overtime work.

Outcome variables

The main outcome variables were self-reported health problems among grain handlers within the previous 14 days. Health problems were grouped into broad anatomical or system-based symptom categories commonly used in occupational health assessments: respiratory, eye, skin, musculoskeletal, and gastrointestinal problems, as well as accidental injuries. Respiratory problems included cough, shortness of breath, chest tightness, and a runny nose. Eye problems included eye redness, watery eyes, and a burning sensation in the eyes. Skin problems included itching and rash. Musculoskeletal problems included pain, numbness, or discomfort in different body regions, including the neck, shoulder, elbow, wrist/hand, upper back, lower back, hip/thigh, knee, ankle, or foot. Gastrointestinal problems included abdominal pain, anorexia, and constipation. For each category, participants were classified as having a health problem if they reported at least one symptom within that category during the previous 14 days. These outcome categories, therefore, represented self-reported symptom prevalence rather than clinically confirmed disease prevalence. Symptom severity, duration, chronicity, and clinical diagnosis were not assessed.

Independent variables

The independent variables included socio-demographic and work-related characteristics. Age was categorized into 19-38 years, 39-58 years, and ≥59 years. Educational qualification was categorized as no formal education/signature only, primary education, and secondary education. Monthly income was categorized as ≤10000 BDT and >10000 BDT. Work experience was categorized as ≤10 years and >10 years. Weekly working hours were categorized as ≤48 hours/week and >48 hours/week. Section of job was categorized as drying, boiling, husking, and loading/unloading.

Statistical analysis

Data were checked for completeness, cleaned, and analyzed using Statistical Package for the Social Sciences (SPSS) version 26.0. Descriptive statistics were used to summarize socio-demographic characteristics, work-related conditions, and reported health problems. Categorical variables were presented as frequencies and percentages. Continuous variables, such as age, monthly income, and weekly working hours, were summarized using means and standard deviations. Bivariable analysis was first performed to examine the association between socio-demographic and work-related variables and each health problem. Crude odds ratios (COR), 95% confidence intervals (CI), and p-values were estimated using logistic regression. Chi-square or Fisher’s exact test was also used where appropriate to compare proportions. Multivariable logistic regression analysis was then performed separately for each major health outcome: respiratory problems, eye problems, skin problems, musculoskeletal problems, and gastrointestinal problems. Adjusted odds ratios (AOR), 95% CI, and p-values were reported. Variables were selected for multivariable models based on conceptual relevance to occupational health outcomes, previous evidence, bivariable findings, biological plausibility, and model stability. Sparse cells and potential collinearity were also considered. Overtime was not included in the adjusted models because it was collinear with weekly working hours. The number of outcome events was 227 for respiratory problems, 73 for eye problems, 48 for skin problems, 189 for musculoskeletal problems, and 49 for gastrointestinal problems. Therefore, models for skin and gastrointestinal problems had relatively fewer events, and the adjusted estimates from these models should be interpreted cautiously. A p-value of <0.05 was considered statistically significant.

Ethical considerations

Ethical approval was obtained from the Institutional Review Board of the National Institute of Preventive and Social Medicine (NIPSOM), Dhaka, Bangladesh (Memo No: NIPSOM/IRB/2021/18, dated 13/12/2021). Informed consent was obtained from all participants before data collection. Participation was voluntary, and respondents were informed about the purpose of the study. Confidentiality of all information was maintained, and data were used only for research purposes.

## Results

Socio-demographic characteristics

Table 1 presents the socio-demographic characteristics of the respondents. The mean age was 38.04±11.01 years, with a range of 19-65 years. Most respondents were male (243 (83.2%)), Muslim (282 (96.6%)), and married (255 (87.3%)). Nearly half had no formal education or could only provide a signature (144 (49.3%)), while 133 (45.5%) had primary education and 15 (5.1%) had secondary education. Most respondents lived in tin-shed houses (262 (89.7%)). The mean monthly income was BDT 10113.01±2891.97, and 189 (64.7%) had a monthly income of ≤BDT 10000. More than half of the respondents were smokers (153 (52.4%)).

**Table 1:** Socio-demographic characteristics of the grain handlers Data are presented as N (%) for categorical variables and mean±SD (range) for continuous variables. Percentages were calculated using the total sample size of 292. BDT, Bangladeshi Taka; SD, standard deviation

Characteristic	Category	N (%) or mean±SD; range
Age, years	19-38	144 (49.3)
	39-58	136 (46.6)
	≥59	12 (4.1)
		38.04±11.01; 19-65
Gender	Male	243 (83.2)
	Female	49 (16.8)
Religion	Muslim	282 (96.6)
	Hindu	10 (3.4)
Educational qualification	No formal education/signature only	144 (49.3)
	Primary education	133 (45.5)
	Secondary education	15 (5.1)
Marital status	Unmarried	37 (12.7)
	Married	255 (87.3)
Family members	≤4	119 (40.8)
	>4	173 (59.2)
Number of family members		4.84±1.41; 2-9
Housing type	Semi-pucca	30 (10.3)
	Tin-shed	262 (89.7)
Monthly income, BDT	≤10000	189 (64.7)
	>10000	103 (35.3)
		10113.01±2891.97; 6000-18000
Smoking habit	Non-smoker	139 (47.6)
	Smoker	153 (52.4)

Work-related characteristics

Table 2 presents the work-related characteristics of the respondents. Nearly half of the respondents had more than 10 years of work experience (142 (48.6%)). Regarding the section of job, 103 (35.3%) worked in the drying section, 74 (25.3%) in loading/unloading, 58 (19.9%) in husking, and 57 (19.5%) in boiling. Most respondents worked more than 48 hours per week (252 (86.3%)), and the same number reported overtime work (252 (86.3%)).

**Table 2:** Work-related characteristics of the grain handlers Data are presented as N (%) for categorical variables and mean±SD; range for continuous variables. Percentages were calculated using the total sample size of 292. SD, standard deviation

Characteristic	Category	N (%) or mean±SD; range
Working experience, years	≤10 years	150 (51.4)
	>10 years	142 (48.6)
		12.69±8.96; 1-40
Working section	Drying	103 (35.3)
	Boiling	57 (19.5)
	Husking	58 (19.9)
	Loading/unloading	74 (25.3)
Weekly working hours	≤48 hours/week	40 (13.7)
	>48 hours/week	252 (86.3)
Weekly working hours		65.94±12.72; 40-84
Overtime work	Yes	252 (86.3)
	No	40 (13.7)

Prevalence of health problems

**Figure 1:**
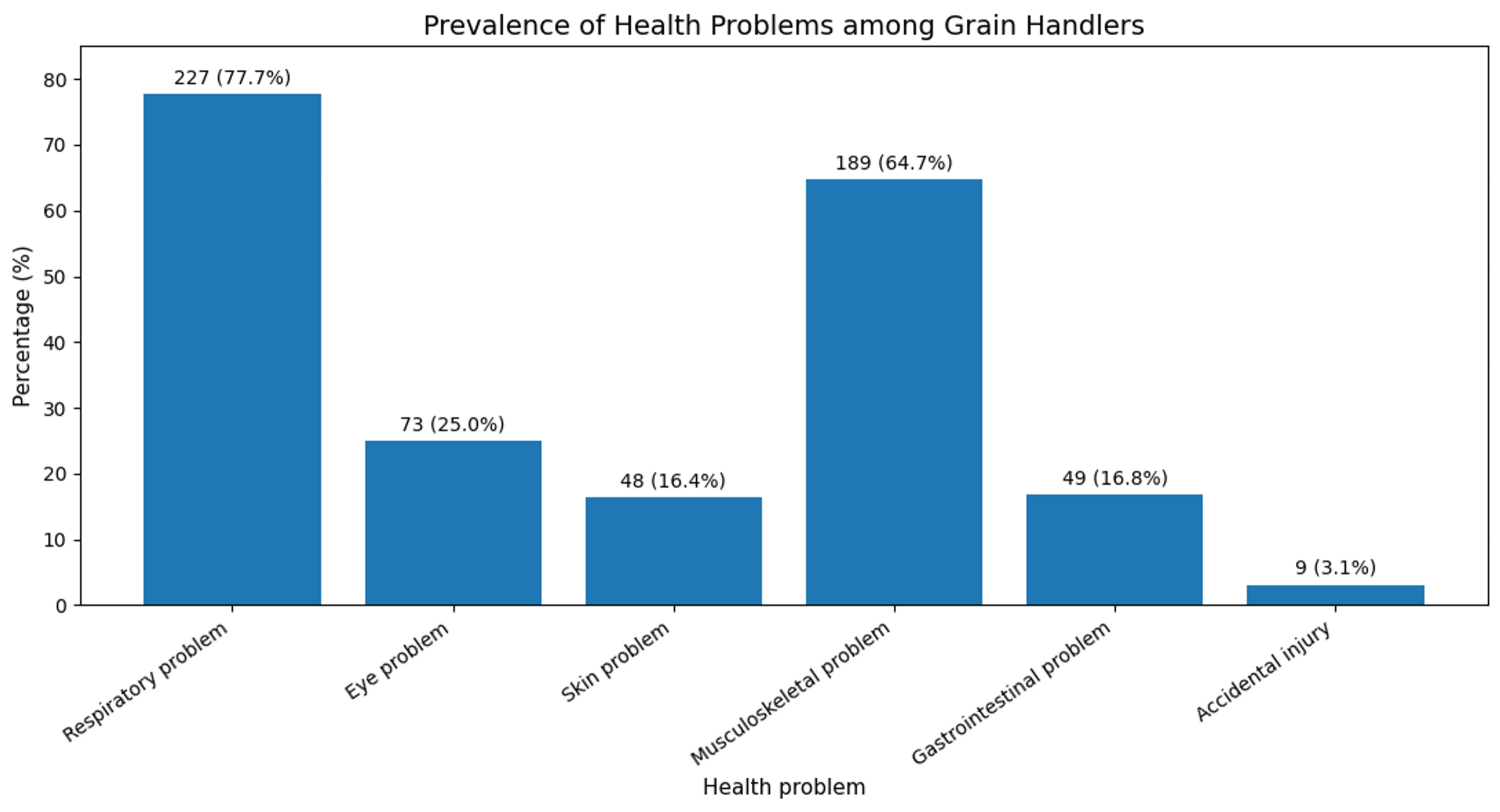
Health problems among the grain handlers

Health problems were common among the grain handlers. Respiratory problems were the most frequently reported, affecting 227 workers (77.7%). Musculoskeletal problems were reported by 189 workers (64.7%), followed by eye problems in 73 (25.0%), gastrointestinal problems in 49 (16.8%), and skin problems in 48 (16.4%). Accidental injuries were reported by nine workers (3.1%) (Figure 1).

Among specific symptoms (Table 3), cough was the most common respiratory symptom, reported by 203 workers (69.5%), followed by chest tightness or discomfort in 95 (32.5%), shortness of breath in 52 (17.8%), and runny nose in 33 (11.3%). Among musculoskeletal symptoms, lower back pain was the most common, reported by 107 workers (36.6%), followed by ankle/foot pain in 68 (23.3%), knee pain in 66 (22.6%), and neck pain in 51 (17.5%). Eye redness was reported by 65 workers (22.3%), watery eyes by 44 (15.1%), and a burning sensation of the eyes by 17 (5.8%). Itching was reported by 48 workers (16.4%), while skin rash was reported by four (1.4%). Among gastrointestinal symptoms, abdominal pain was reported by 29 workers (9.9%), anorexia by 24 (8.2%), and constipation by five (1.7%).

**Table 3:** Symptoms reported by the grain handlers Data are presented as N (%). Percentages were calculated using the total sample size of 292.

Health problem category	Symptom	Yes, N (%)
Respiratory symptoms	Cough	203 (69.5)
	Shortness of breath	52 (17.8)
	Chest tightness/discomfort	95 (32.5)
	Running nose	33 (11.3)
Eye symptoms	Red eye	65 (22.3)
	Watering from eye	44 (15.1)
	Eye burning	17 (5.8)
Skin symptoms	Itching in any body part	48 (16.4)
	Skin rash	4 (1.4)
Musculoskeletal symptoms	Neck pain/discomfort	51 (17.5)
	Shoulder pain/discomfort	50 (17.1)
	Elbow pain/discomfort	11 (3.8)
	Hand/wrist joint pain/discomfort	38 (13.0)
	Upper back pain/discomfort	11 (3.8)
	Lower back pain/discomfort	107 (36.6)
	Hip/thigh pain/discomfort	16 (5.5)
	Knee joint pain/discomfort	66 (22.6)
	Ankle/foot pain/discomfort	68 (23.3)
Gastrointestinal symptoms	Abdominal pain	29 (9.9)
	Anorexia/distaste	24 (8.2)
	Constipation	5 (1.7)

Factors associated with respiratory problems

In bivariable analysis, no socio-demographic or work-related factor was significantly associated with respiratory problems. Although respiratory symptoms were highly prevalent among grain handlers, the odds of respiratory problems did not vary significantly by age, gender, education, marital status, income, smoking status, work experience, section of job, or weekly working hours (Table 4).

**Table 4:** Factors associated with respiratory problems among grain handlers Data are presented as N (%). COR and AOR are presented with 95% CIs. P-values <0.05 were considered statistically significant; p<0.001 indicates strong statistical significance. AOR, adjusted odds ratio; BDT, Bangladeshi Taka; CI, confidence interval; COR, crude odds ratio

Variable	Category	Total, N	Problem present, N (%)	COR (95% CI)	P-value	AOR (95% CI)	P-value
Age group (years)	Overall p=0.107						
	19-38	144	105 (72.9)	1.00	-	1.00	-
	39-58	136	111 (81.6)	1.65 (0.93-2.91)	0.085	1.50 (0.81-2.77)	0.200
	≥59	12	11 (91.7)	4.09 (0.51-32.70)	0.185	3.80 (0.47-30.86)	0.212
Gender	Overall p=0.473						
	Male	243	187 (77.0)	1.00	-	1.00	-
	Female	49	40 (81.6)	1.33 (0.61-2.91)	0.474	1.25 (0.56-2.77)	0.582
Religion	Overall p=0.467						
	Muslim	282	218 (77.3)	1.00	-	-	-
	Hindu	10	9 (90.0)	2.64 (0.33-21.25)	0.361	-	-
Educational qualification	Overall p=0.271						
	No formal/signature only	144	116 (80.6)	1.00	-	-	-
	Primary	133	98 (73.7)	0.68 (0.38-1.19)	0.174	-	-
	Secondary	15	13 (86.7)	1.57 (0.33-7.35)	0.568	-	-
Marital status	Overall p=0.111						
	Unmarried	37	25 (67.6)	1.00	-	1.00	-
	Married	255	202 (79.2)	1.83 (0.86-3.88)	0.115	1.37 (0.60-3.13)	0.449
Family members	Overall p=0.315						
	≤4	119	89 (74.8)	1.00	-	-	-
	>4	173	138 (79.8)	1.33 (0.76-2.32)	0.316	-	-
Type of house	Overall p=0.282						
	Semi-pucca	30	21 (70.0)	1.00	-	-	-
	Tin-shed	262	206 (78.6)	1.58 (0.68-3.63)	0.285	-	-
Monthly income (BDT)	Overall p=0.389						
	≤10000	189	144 (76.2)	1.00	-	-	-
	>10000	103	83 (80.6)	1.30 (0.72-2.34)	0.389	-	-
Smoking habit	Overall p=0.562						
	Non-smoker	139	106 (76.3)	1.00	-	-	-
	Smoker	153	121 (79.1)	1.18 (0.68-2.04)	0.562	-	-
Working experience	Overall p=0.310						
	≤10 years	150	113 (75.3)	1.00	-	-	-
	>10 years	142	114 (80.3)	1.33 (0.76-2.32)	0.310	-	-
Section of job	Overall p=0.417						
	Drying	103	83 (80.6)	1.00	-	-	-
	Boiling	57	42 (73.7)	0.67 (0.31-1.45)	0.314	-	-
	Husking	58	48 (82.8)	1.16 (0.50-2.67)	0.734	-	-
	Loading/unloading	74	54 (73.0)	0.65 (0.32-1.32)	0.234	-	-
Weekly working hours	Overall p=0.969						
	≤48 hours/week	40	31 (77.5)	1.00	-	-	-
	>48 hours/week	252	196 (77.8)	1.02 (0.46-2.26)	0.969	-	-
Overtime	Overall p=0.969						
	No	40	31 (77.5)	1.00	-	-	-
	Yes	252	196 (77.8)	1.02 (0.46-2.26)	0.969	-	-

Factors associated with eye problems

In bivariable analysis, eye problems were significantly associated with weekly working hours and overtime work. In the multivariable model, section of job and weekly working hours remained significantly associated with eye problems. Compared with workers in the drying section, workers in the boiling section had lower odds of eye problems (AOR=0.40, 95% CI: 0.17-0.96; p=0.039). Workers who worked >48 hours per week also had lower odds of eye problems than those who worked ≤48 hours per week (AOR=0.33, 95% CI: 0.16-0.68; p=0.003) (Table 5). Because this latter finding is counterintuitive, it should be interpreted cautiously and not as evidence that longer working hours protect against eye problems.

**Table 5:** Factors associated with eye problems among grain handlers Data are presented as N (%). COR and AOR are presented with 95% CI. P-values <0.05 were considered statistically significant; p<0.001 indicates strong statistical significance. AOR, adjusted odds ratio; BDT, Bangladeshi Taka; CI, confidence interval; COR, crude odds ratio

Variable	Category	Total, N	Problem present, N (%)	COR (95% CI)	P-value	AOR (95% CI)	P-value
Age group (years)	Overall p=0.102						
	19-38	144	29 (20.1)	1.00	-	1.00	-
	39-58	136	39 (28.7)	1.59 (0.92-2.77)	0.097	1.40 (0.69-2.84)	0.356
	≥59	12	5 (41.7)	2.83 (0.84-9.57)	0.094	2.39 (0.61-9.38)	0.212
Gender	Overall p=0.086						
	Male	243	56 (23.0)	1.00	-	1.00	-
	Female	49	17 (34.7)	1.77 (0.92-3.43)	0.089	1.01 (0.24-4.27)	0.989
Religion	Overall p=0.071						
	Muslim	282	73 (25.9)	1.00	-	-	-
	Hindu	10	0 (0.0)	0.14 (0.01-2.35)	0.170	-	-
Educational qualification	Overall p=0.295						
	No formal/signature only	144	41 (28.5)	1.00	-	-	-
	Primary	133	30 (22.6)	0.73 (0.42-1.26)	0.261	-	-
	Secondary	15	2 (13.3)	0.39 (0.08-1.79)	0.224	-	-
Marital status	Overall p=0.919						
	Unmarried	37	9 (24.3)	1.00	-	-	-
	Married	255	64 (25.1)	1.04 (0.47-2.33)	0.919	-	-
Family members	Overall p=0.731						
	≤4	119	31 (26.1)	1.00	-	-	-
	>4	173	42 (24.3)	0.91 (0.53-1.56)	0.731	-	-
Type of house	Overall p=0.180						
	Semi-pucca	30	4 (13.3)	1.00	-	1.00	-
	Tin-shed	262	69 (26.3)	2.32 (0.78-6.90)	0.129	1.83 (0.59-5.71)	0.297
Monthly income (BDT)	Overall p=0.437						
	≤10000	189	50 (26.5)	1.00	-	-	-
	>10000	103	23 (22.3)	0.80 (0.45-1.41)	0.437	-	-
Smoking habit	Overall p=0.155						
	Non-smoker	139	40 (28.8)	1.00	-	1.00	-
	Smoker	153	33 (21.6)	0.68 (0.40-1.16)	0.157	0.89 (0.46-1.74)	0.733
Working experience	Overall p=0.137						
	≤10 years	150	32 (21.3)	1.00	-	1.00	-
	>10 years	142	41 (28.9)	1.50 (0.88-2.55)	0.138	1.07 (0.53-2.15)	0.846
Section of job	Overall p=0.091						
	Drying	103	29 (28.2)	1.00	-	1.00	-
	Boiling	57	10 (17.5)	0.54 (0.24-1.22)	0.138	0.40 (0.17-0.96)	0.039
	Husking	58	20 (34.5)	1.34 (0.67-2.68)	0.403	0.94 (0.22-3.93)	0.929
	Loading/unloading	74	14 (18.9)	0.60 (0.29-1.23)	0.160	0.58 (0.28-1.23)	0.158
Weekly working hours	Overall p<0.001						
	≤48 hours/week	40	19 (47.5)	1.00	-	1.00	-
	>48 hours/week	252	54 (21.4)	0.30 (0.15-0.60)	<0.001	0.33 (0.16-0.68)	0.003
Overtime	Overall p<0.001						
	No	40	19 (47.5)	1.00	-	-	-
	Yes	252	54 (21.4)	0.30 (0.15-0.60)	<0.001	-	-

Factors associated with skin problems

In bivariable analysis, skin problems were significantly associated with gender (p<0.001), educational qualification (p=0.004), monthly income (p=0.050), smoking habit (p=0.004), and section of job (p=0.001). After adjustment in the multivariable logistic regression model, educational qualification remained significantly associated with skin problems. Workers with primary education had lower odds of skin problems compared with those with no formal education/signature only (AOR=0.39, 95% CI: 0.17-0.88; p=0.024). Other variables that were significant in the bivariable analysis did not remain statistically significant after adjustment (Table 6).

**Table 6:** Factors associated with skin problems among grain handlers Data are presented as N (%). COR and AOR are presented with 95% CI. P-values <0.05 were considered statistically significant; p<0.001 indicates strong statistical significance. AOR, adjusted odds ratio; BDT, Bangladeshi Taka; CI, confidence interval; COR, crude odds ratio

Variable	Category	Total, N	Problem present, N (%)	COR (95% CI)	P-value	AOR (95% CI)	P-value
Age group (years)	Overall p=0.715						
	19-38	144	23 (16.0)	1.00	-	1.00	-
	39-58	136	22 (16.2)	1.02 (0.54-1.92)	0.963	0.63 (0.30-1.32)	0.222
	≥59	12	3 (25.0)	1.75 (0.44-6.97)	0.425	1.04 (0.22-4.88)	0.962
Gender	Overall p=0.001						
	Male	243	32 (13.2)	1.00	-	1.00	-
	Female	49	16 (32.7)	3.20 (1.58-6.46)	0.001	0.90 (0.21-3.83)	0.891
Religion	Overall p=0.216						
	Muslim	282	45 (16.0)	1.00	-	-	-
	Hindu	10	3 (30.0)	2.26 (0.56-9.06)	0.251	-	-
Educational qualification	Overall p=0.004						
	No formal/signature only	144	34 (23.6)	1.00	-	1.00	-
	Primary	133	12 (9.0)	0.32 (0.16-0.65)	0.002	0.39 (0.17-0.88)	0.024
	Secondary	15	2 (13.3)	0.50 (0.11-2.32)	0.374	0.59 (0.11-3.00)	0.521
Marital status	Overall p=0.363						
	Unmarried	37	8 (21.6)	1.00	-	-	-
	Married	255	40 (15.7)	0.67 (0.29-1.58)	0.365	-	-
Family members	Overall p=0.410						
	≤4	119	17 (14.3)	1.00	-	-	-
	>4	173	31 (17.9)	1.31 (0.69-2.49)	0.411	-	-
Type of house	Overall p=0.191						
	Semi-pucca	30	2 (6.7)	1.00	-	1.00	-
	Tin-shed	262	46 (17.6)	2.98 (0.69-12.96)	0.145	2.50 (0.54-11.63)	0.244
Monthly income (BDT)	Overall p=0.050						
	≤10000	189	37 (19.6)	1.00	-	1.00	-
	>10000	103	11 (10.7)	0.49 (0.24-1.01)	0.053	0.93 (0.40-2.19)	0.877
Smoking habit	Overall p=0.004						
	Non-smoker	139	32 (23.0)	1.00	-	1.00	-
	Smoker	153	16 (10.5)	0.39 (0.20-0.75)	0.005	0.70 (0.31-1.60)	0.396
Working experience	Overall p=0.671						
	≤10 years	150	26 (17.3)	1.00	-	-	-
	>10 years	142	22 (15.5)	0.87 (0.47-1.63)	0.672	-	-
Section of job	Overall p=0.001						
	Drying	103	17 (16.5)	1.00	-	1.00	-
	Boiling	57	4 (7.0)	0.38 (0.12-1.20)	0.098	0.34 (0.11-1.11)	0.073
	Husking	58	19 (32.8)	2.46 (1.16-5.25)	0.019	1.44 (0.33-6.25)	0.624
	Loading/unloading	74	8 (10.8)	0.61 (0.25-1.51)	0.287	0.62 (0.24-1.58)	0.314
Weekly working hours	Overall p=0.266						
	≤48 hours/week	40	9 (22.5)	1.00	-	-	-
	>48 hours/week	252	39 (15.5)	0.63 (0.28-1.43)	0.269	-	-
Overtime	Overall p=0.266						
	No	40	9 (22.5)	1.00	-	-	-
	Yes	252	39 (15.5)	0.63 (0.28-1.43)	0.269	-	-

Factors associated with musculoskeletal problems

In bivariable analysis, musculoskeletal problems were significantly associated with age group (p<0.001), educational qualification (p=0.001), marital status (p=0.011), type of house (p=0.003), work experience (p=0.013), and section of job (p=0.005). In multivariable analysis, age group and section of job remained significantly associated with musculoskeletal problems. Workers aged 39-58 years had higher odds of musculoskeletal problems compared with those aged 19-38 years (AOR=2.71, 95% CI: 1.35-5.44; p=0.005). Workers engaged in loading/unloading also had higher odds of musculoskeletal problems compared with workers in the drying section (AOR=2.23, 95% CI: 1.10-4.53; p=0.026). These associations may be influenced by cumulative work duration and unmeasured ergonomic exposures. Other factors were not statistically significant after adjustment (Table 7).

**Table 7:** Factors associated with musculoskeletal problems among grain handlers Data are presented as N (%). COR and AOR are presented with 95% CI. P-values <0.05 were considered statistically significant; p<0.001 indicates strong statistical significance. AOR, adjusted odds ratio; BDT, Bangladeshi Taka; CI, confidence interval; COR, crude odds ratio

Variable	Category	Total, N	Problem present, N (%)	COR (95% CI)	P-value	AOR (95% CI)	P-value
Age group (years)	Overall p<0.001						
	19-38	144	75 (52.1)	1.00	-	1.00	-
	39-58	136	106 (77.9)	3.25 (1.93-5.47)	<0.001	2.71 (1.35-5.44)	0.005
	≥59	12	8 (66.7)	1.84 (0.53-6.38)	0.337	1.55 (0.39-6.26)	0.536
Gender	Overall p=0.083						
	Male	243	152 (62.6)	1.00	-	1.00	-
	Female	49	37 (75.5)	1.85 (0.92-3.72)	0.087	1.34 (0.28-6.54)	0.716
Religion	Overall p=0.746						
	Muslim	282	183 (64.9)	1.00	-	-	-
	Hindu	10	6 (60.0)	0.81 (0.22-2.94)	0.751	-	-
Educational qualification	Overall p=0.001						
	No formal/signature only	144	107 (74.3)	1.00	-	1.00	-
	Primary	133	71 (53.4)	0.40 (0.24-0.66)	<0.001	0.58 (0.32-1.06)	0.075
	Secondary	15	11 (73.3)	0.95 (0.29-3.17)	0.935	2.21 (0.59-8.26)	0.237
Marital status	Overall p=0.011						
	Unmarried	37	17 (45.9)	1.00	-	1.00	-
	Married	255	172 (67.5)	2.44 (1.21-4.90)	0.012	1.31 (0.56-3.04)	0.534
Family members	Overall p=0.995						
	≤4	119	77 (64.7)	1.00	-	-	-
	>4	173	112 (64.7)	1.00 (0.61-1.63)	0.995	-	-
Type of house	Overall p=0.003						
	Semi-pucca	30	12 (40.0)	1.00	-	1.00	-
	Tin-shed	262	177 (67.6)	3.12 (1.44-6.78)	0.004	2.27 (0.96-5.35)	0.061
Monthly income (BDT)	Overall p=0.494						
	≤10000	189	125 (66.1)	1.00	-	-	-
	>10000	103	64 (62.1)	0.84 (0.51-1.38)	0.494	-	-
Smoking habit	Overall p=0.139						
	Non-smoker	139	96 (69.1)	1.00	-	1.00	-
	Smoker	153	93 (60.8)	0.69 (0.43-1.13)	0.140	0.92 (0.49-1.70)	0.779
Working experience	Overall p=0.013						
	≤10 years	150	87 (58.0)	1.00	-	1.00	-
	>10 years	142	102 (71.8)	1.85 (1.13-3.01)	0.014	1.01 (0.52-1.97)	0.970
Section of job	Overall p=0.005						
	Drying	103	57 (55.3)	1.00	-	1.00	-
	Boiling	57	32 (56.1)	1.03 (0.54-1.98)	0.922	0.76 (0.37-1.53)	0.439
	Husking	58	44 (75.9)	2.54 (1.24-5.19)	0.011	1.08 (0.23-5.11)	0.924
	Loading/unloading	74	56 (75.7)	2.51 (1.30-4.85)	0.006	2.23 (1.10-4.53)	0.026
Weekly working hours	Overall p=0.268						
	≤48 hours/week	40	29 (72.5)	1.00	-	-	-
	>48 hours/week	252	160 (63.5)	0.66 (0.31-1.38)	0.270	-	-
Overtime	Overall p=0.268						
	No	40	29 (72.5)	1.00	-	-	-
	Yes	252	160 (63.5)	0.66 (0.31-1.38)	0.270	-	-

Factors associated with gastrointestinal problems

In bivariable analysis, most socio-demographic and work-related factors were not significantly associated with gastrointestinal problems. In multivariable analysis, weekly working hours remained significantly associated with gastrointestinal problems. Workers who worked >48 hours per week had higher odds of gastrointestinal problems compared with those who worked ≤48 hours per week (AOR=4.05, 95% CI: 1.16-14.15; p=0.029). The wide CI indicates statistical imprecision; therefore, this association should be interpreted cautiously (Table 8).

**Table 8:** Factors associated with gastrointestinal problems among grain handlers Data are presented as N (%). COR and AOR are presented with 95% CI. P-values <0.05 were considered statistically significant; p<0.001 indicates strong statistical significance. AOR, adjusted odds ratio; BDT, Bangladeshi Taka; CI, confidence interval; COR, crude odds ratio

Variable	Category	Total, N	Problem present, N (%)	COR (95% CI)	P-value	AOR (95% CI)	P-value
Age group (years)	Overall p=0.502						
	19-38	144	22 (15.3)	1.00	-	1.00	-
	39-58	136	26 (19.1)	1.31 (0.70-2.45)	0.395	1.14 (0.57-2.26)	0.712
	≥59	12	1 (8.3)	0.50 (0.06-4.10)	0.522	0.47 (0.05-4.04)	0.488
Gender	Overall p=0.456						
	Male	243	39 (16.0)	1.00	-	1.00	-
	Female	49	10 (20.4)	1.34 (0.62-2.91)	0.458	1.95 (0.32-11.81)	0.467
Religion	Overall p=1.000						
	Muslim	282	48 (17.0)	1.00	-	-	-
	Hindu	10	1 (10.0)	0.54 (0.07-4.38)	0.565	-	-
Educational qualification	Overall p=0.529						
	No formal/signature only	144	26 (18.1)	1.00	-	-	-
	Primary	133	22 (16.5)	0.90 (0.48-1.68)	0.739	-	-
	Secondary	15	1 (6.7)	0.32 (0.04-2.58)	0.287	-	-
Marital status	Overall p=0.161						
	Unmarried	37	3 (8.1)	1.00	-	1.00	-
	Married	255	46 (18.0)	2.49 (0.73-8.47)	0.143	2.65 (0.71-9.86)	0.146
Family members	Overall p=0.199						
	≤4	119	24 (20.2)	1.00	-	-	-
	>4	173	25 (14.5)	0.67 (0.36-1.24)	0.201	-	-
Type of house	Overall p=0.193						
	Semi-pucca	30	2 (6.7)	1.00	-	1.00	-
	Tin-shed	262	47 (17.9)	3.06 (0.70-13.30)	0.136	3.23 (0.71-14.61)	0.128
Monthly income (BDT)	Overall p=0.223						
	≤10000	189	28 (14.8)	1.00	-	-	-
	>10000	103	21 (20.4)	1.47 (0.79-2.75)	0.225	-	-
Smoking habit	Overall p=0.466						
	Non-smoker	139	21 (15.1)	1.00	-	-	-
	Smoker	153	28 (18.3)	1.26 (0.68-2.34)	0.466	-	-
Working experience	Overall p=0.496						
	≤10 years	150	23 (15.3)	1.00	-	-	-
	>10 years	142	26 (18.3)	1.24 (0.67-2.29)	0.497	-	-
Section of job	Overall p=0.076						
	Drying	103	16 (15.5)	1.00	-	1.00	-
	Boiling	57	15 (26.3)	1.94 (0.88-4.30)	0.102	1.89 (0.83-4.32)	0.130
	Husking	58	11 (19.0)	1.27 (0.55-2.96)	0.576	0.59 (0.10-3.45)	0.557
	Loading/unloading	74	7 (9.5)	0.57 (0.22-1.46)	0.240	0.44 (0.17-1.17)	0.100
Weekly working hours	Overall p=0.112						
	≤48 hours/week	40	3 (7.5)	1.00	-	1.00	-
	>48 hours/week	252	46 (18.3)	2.75 (0.81-9.32)	0.103	4.05 (1.16-14.15)	0.029
Overtime	Overall p=0.112						
	No	40	3 (7.5)	1.00	-	-	-
	Yes	252	46 (18.3)	2.75 (0.81-9.32)	0.103	-	-

## Discussion

This cross-sectional study found a high burden of self-reported health problems among grain handlers working in rice mills in Bangladesh. Respiratory problems were the most frequently reported health problems, followed by musculoskeletal problems. Eye, gastrointestinal, and skin problems were also reported. These findings suggest that grain handlers experience multiple health complaints in a work environment characterized by dust, smoke, heat, prolonged working hours, repetitive movements, awkward postures, and heavy manual workload. However, because of the cross-sectional design and reliance on self-reported symptoms, the findings should be interpreted as associations and not as evidence of causality.

Respiratory problems were highly prevalent, with cough being the most common symptom. This may be related to frequent exposure to rice dust, husk particles, bran, silica, fungi, bacteria, endotoxins, and other contaminants during drying, husking, loading, unloading, and milling [6,11]. The prevalence in this study was higher than that reported by Ansari et al. among rice mill workers in Bangladesh, where 34.0% had chronic respiratory symptoms [12]. This difference may be due to differences in symptom definition, recall period, exposure level, work setting, and whether symptoms were self-reported or clinically assessed. The broad symptom definition used in the present study may have increased the reported prevalence because even isolated or mild symptoms were included.

In adjusted analysis, no socio-demographic or work-related factor was significantly associated with respiratory problems. This may be because respiratory hazards were common across most sections of job, resulting in limited variation in exposure between worker groups. Smoking is also an important potential contributor to respiratory symptoms; although smoking status was included in the analysis, detailed smoking exposure, such as pack-years, and its interaction with dust exposure could not be assessed. The use of self-reported symptoms without spirometry, clinical examination, or dust exposure measurements may also have limited the ability to identify specific risk factors.

Musculoskeletal problems were reported by nearly two-thirds of workers. Lower back pain was the most common symptom, followed by ankle/foot pain, knee pain, and neck pain. This pattern is consistent with the physical demands of grain handling, including lifting and carrying heavy sacks, prolonged standing, bending, and repetitive movements. Similar findings have been reported in previous studies among rice mill workers [13,14]. In the present study, workers aged 39-58 years had significantly higher odds of musculoskeletal problems than those aged 19-38 years, and loading/unloading workers had higher odds than drying workers. These findings are plausible given the physical workload involved; however, residual confounding from work duration, task history, and unmeasured ergonomic exposures may still exist.

Eye problems were reported by one-fourth of the respondents. Redness, watering, and a burning sensation of the eyes may be related to exposure to dust, smoke, heat, and poor ventilation. Workers in the boiling section had lower odds of eye problems compared with those in the drying section, which may reflect greater airborne dust exposure during drying activities. However, this explanation remains speculative because environmental dust levels were not measured. Workers who worked more than 48 hours per week also had lower odds of eye problems compared with those working ≤48 hours per week. This counterintuitive finding should be interpreted cautiously and should not be considered evidence that longer working hours are protective. It may reflect residual confounding, selection effects, reporting bias, the healthy worker effect, or differences in task assignment. Eye problems such as redness, watering, and a burning sensation may be related to continuous exposure to dust, smoke, heat, and poor ventilation in the rice mill environment. Similar ocular complaints among rice mill workers were reported in previous studies [14,15].

Skin problems were reported by 16.4% of workers, mainly itching and rash. These symptoms may result from direct contact with grain dust, husk, bran, sweat, heat, and contaminated surfaces. In adjusted analysis, workers with primary education had lower odds of skin problems compared with those with no formal education/signature only. This may reflect better awareness of hygiene and protective practices among workers with some formal education, although residual confounding cannot be excluded. Comparable dermatological complaints among agricultural and rice mill workers were also documented in earlier studies [16,17].

Gastrointestinal problems were reported by 16.8% of workers. These symptoms may be related to irregular meals, dehydration, poor workplace sanitation, and long working hours. In multivariable analysis, working more than 48 hours per week was associated with higher odds of gastrointestinal problems. However, the CI was wide, indicating statistical imprecision. This finding suggests that prolonged working time may affect eating habits, rest, hydration, and overall health. Similar gastrointestinal morbidities among rice mill workers were also observed in previous studies [14,15]. Longer working hours may adversely affect eating patterns, hydration, and adequate rest.

This study has several limitations. The cross-sectional design prevents causal inference and does not allow determination of whether symptoms began before or after employment. Health problems were self-reported within a 14-day recall period and may be affected by recall or reporting bias. The outcomes represented symptom prevalence rather than clinically confirmed disease prevalence. The broad symptom definitions may have overestimated the burden of health problems because mild, isolated, or transient symptoms were classified in the same way as more clinically significant conditions. Objective assessments such as spirometry, clinical examination, personal dust monitoring, heat measurement, ventilation assessment, air quality monitoring, and structured ergonomic evaluation were not performed. Therefore, associations between specific workplace exposures and reported symptoms could not be directly quantified. The study may also be affected by the healthy worker survivor effect because workers who had left rice mill work due to illness, dust exposure, or heavy physical workload were not included. Smoking intensity and the interaction between smoking and dust exposure could not be assessed. Unequal representation across participant groups may have introduced selection bias and should be considered when interpreting the study findings. Finally, the study was conducted in rice mills in only one upazila, which may limit generalizability to grain handlers in other regions of Bangladesh where mill technology, work organization, socioeconomic conditions, exposure levels, and occupational safety practices may differ.

## Conclusions

Grain handlers in Bangladesh reported a high burden of occupational health-related symptoms, particularly respiratory and musculoskeletal symptoms. Selected socio-demographic and work-related factors, including age, education, section of job, and weekly working hours, were associated with some self-reported health symptom categories. These findings should be interpreted cautiously because of the cross-sectional design, reliance on self-reported symptoms, absence of objective clinical or exposure assessments, and limited generalizability from one study area. These findings highlight the need for improved occupational safety measures, including dust control, better ventilation, ergonomic interventions, scheduled rest periods, health education, appropriate PPE, and regular health screening.
